# Is the likelihood of spousal violence lower or higher among childless women? Evidence from Nigeria demographic and health surveys

**DOI:** 10.1186/s12905-018-0514-3

**Published:** 2018-01-16

**Authors:** Bola Lukman Solanke, Adeleke Luqman Bisiriyu, Amos Oyedokun

**Affiliations:** Department of Demography and Social Statistics, Obafemi Awolowo University, Ile-Ife, Nigeria

**Keywords:** Childlessness, Spousal violence, Women, Infertility, Children, Nigeria

## Abstract

**Background:**

Few studies have been able to determine whether the likelihood of spousal violence is higher or lower among childless women compared with women who have children. This is because most studies linking childlessness and spousal violence were either qualitative or were conducted among childless women attending fertility clinics. In the fewer quantitative studies that linked childlessness and spousal violence, results are mixed and yet to be verified in Nigeria using nationally representative sample data. The current study addresses this knowledge gap by raising the research question: is the likelihood of spousal violence lower or higher among childless women?

**Methods:**

The study analysed data from 2008 and 2013 Nigeria Demographic and Health Surveys. Only women aged 35–49 years are included in the analysis. The outcome variable was spousal violence, while the key explanatory variable was parity status categorised into childless, have only one child, and have two or more children. Selected individual-level and community-level variables were included as additional explanatory variables. The multilevel mixed-effects logistic regression analysis was applied in four nested models using Stata 12.

**Results:**

In Model 1, result show 57% more likelihood of spousal violence among women who have two or more children compared with childless women (OR = 1.570: CI: 1.074–2.294). In Model 2, women who have two or more children were 52.3% more likely to experience spousal violence compared with childless women (OR = 1.523; CI: 1.037–2.247). In Model 3, the likelihood of spousal violence was 67.2% higher among women who have two or more children compared with childless women (OR = 1.672; CI: 1.140–2.452). In the full model, women who have two or more children were 50.8% more likely to experience spousal violence compared with childless women (OR = 1.508; CI: 1.077–2.234). The Intra-Class Correlation (ICC) provides evidence to support community contributions to prevalence of spousal violence.

**Conclusions:**

The likelihood of spousal violence is lower among childless women in Nigeria. Causes of spousal violence against women cut across individual, family, and community characteristics irrespective of childlessness or number of children. Current Behaviour Change Communication should be strengthened by adequate enforcement of the newly enacted Violence Against Persons (Prohibition) Act of 2015.

## Background

Childlessness in this study refers to inability to have ever born a child, and was measured from women’s parity (number of children previously born alive). This connotes primary infertility (never having had a live birth) and distinct from secondary infertility (inability to have another live birth after a previous live birth). The study is however not focusing on the epidemiologic or clinical aspects of childlessness but attempts to associate being childless to women’s experience of spousal violence. In Africa, particularly sub-Saharan Africa, fertility is highly valued and celebrated. The cultural conditions promoting high fertility in most parts of sub-Saharan Africa earlier described [1] remain rooted in several African communities and not only account for some of the reasons why fertility on the average remain high in several African countries [2], it also account for some of the reasons why contraceptive use remain low [3] in spite of the implementation of national family planning programmes in many parts of sub-Saharan Africa [4]. Hence, childlessness is a cultural aberration in sub-Saharan Africa and widely unacceptable in most African communities [5–7]. In many African communities, childless women often suffer serious social and economic disadvantages and sometimes faces outright discrimination, humiliation and condemnation [8] which has not only made some childless women to become desperate to have a child at all cost, but to also patronise ‘baby factories’ to exchange money for children [9].

The proportion of childless women though unknown in many communities, is usually not expected to be substantial which may be the reason why childlessness has remain a neglected health problem in sub-Saharan Africa [10]. Estimates of the prevalence of infertility and childlessness across the world have not been uniform. In an earlier attempt using Demographic and Health Survey (DHS) datasets from 47 developing countries, it was estimated that in 2002, more than 186 million ever-married women in developing countries with the exclusion of China were affected either by primary or secondary infertility [11]. Recent attempts estimated that between 48.5 and 72.4 million women or couples are affected by infertility [12, 13]. In Nigeria, numerous studies have provided evidence of varying degrees of infertility and childlessness among women, men and couples in the country [14, 15]. Childlessness may result from preventable causes such as sexually transmitted diseases, infectious and parasitic diseases, poor health behaviour such as unsafe abortion, advancing maternal age, socio-cultural factors such as female genital cutting, multiple sexual partners, early marriage, and early sexual debut, and a number of gynaecological factors [14, 16].

The psychological and social consequences associated with childlessness include but not limited to marital conflict [17]; psychiatric morbidity [18]; psychological distress [19]; sexual dysfunction [20]; and spousal violence [21, 22]. Though, all the consequences affect sexual and reproductive health of childless women, but increasing number of recent studies have focus attention on the relationship between childlessness and spousal violence [23–25]. Spousal violence which is also known as Intimate Partner Violence (IPV), refers to all forms of physical, sexual or psychological harm that occurs between couples or intimate partners [26]. Evidence abounds that spousal violence is prevalent with deleterious effects on women’s health across the world [27–29]. It is more worrisome that spousal violence is also widely reported against pregnant women [30–32].

However, in spite of increasing studies linking childlessness and spousal violence across the world [21–25, 33–35], only few studies have been able to determine whether the likelihood of spousal violence is higher or lower among childless women compared with women who have children. Two reasons account for this knowledge gap. Firstly, many of the studies are conducted among infertile women attending fertility clinics [21, 23–25, 33]. These studies are unable to factually determine whether spousal violence was higher or lower among childless women because the samples analysed do not have a comparison group of fertile women. Secondly, other studies have been mainly qualitative exploring cultural perception and interpretation of childlessness or infertility [7, 36].

In the fewer quantitative studies that included both childless women and women who have children, results are mixed. In a Tanzanian study, both childless women and women with high parity had elevated risk of spousal violence [37], but in a study conducted in India, childless women had lower likelihood of spousal violence [38]. This finding was contradicted by two Indian studies based on 2005/2006 National Family Health Survey (NFHS-3) data. The first study reported 77.8% prevalence of spousal physical/sexual violence among childless women compared with 6.1% among women who have children [22], while the second study reported the same 9.6% prevalence of forced sex among women who have children and childless women, though other types of violence were slightly higher among childless women in the study [39], but the differences were marginal. In a recent Nigerian study, it was reported that childlessness was not a significant predictor of spousal abuse [34]. The study was however, not nationally representative.

To our best knowledge, these findings have not been factually updated in Nigeria particularly using nationally representative sample data. The current study addresses this knowledge gap by raising the research question: is the likelihood of spousal violence lower or higher among childless women? The objective of the study was therefore to examine the association of parity and spousal violence. This was with the view to promoting the reproductive health and rights of childless women by providing additional information on an underlying factor of spousal violence in Nigeria. The country was selected for the study because being the most populous country in Africa, the absolute number of women affected by both childlessness and spousal violence is more likely to be higher than in any other African country. The socio-ecological model which asserts that there are multiple influences on health behaviours [40] provides the theoretical underpinning of the study.

## Methods

### Context

The geographic location of the study is Nigeria, the most populous country in Africa and the seventh most populous country in the world [41]. National statistics of childlessness are hardly available in Nigeria. Studies in the country have however provided varying evidence on the prevalence, causes, consequences and management of infertility and childlessness in the country [7, 36, 42]. In spite of lack of national statistics, a number of national policies and intervention addressing infertility and childlessness exist in the country. The current National Policy on Population for Sustainable Development expresses commitment to the provision of family planning services that addresses the fertility challenges of sterile and sub-fertile individuals and couples in the country [43]. The 2004 Revised National Health Policy based on primary health care also emphasise services that could help individuals and couples including childless women and couples to secure desired pregnancies [44].

The National Family Planning/Reproductive Health Service Protocols also provides sufficient counselling strategies for infertile and childless individuals and couples in the country [45]. The Service Protocol however noted that assisted reproductive techniques used in the management of infertility such as fertility medication, in-vitro fertilization, artificial insemination, assisted hatching, cryopreservation, sex selection, surrogacy and reproductive surgery are not affordable and accessible to majority of infertile and childless couples, leaving child adoption as the most viable option out of childlessness though attitude to child adoption is still poor in the country [46]. How the challenges of childlessness modulate the likelihood of spousal violence has not been well documented in Nigeria. But programmes and legislations do exist for reducing the prevalence of gender-based violence in the country. Behaviour Change Communication (BCC) is one of the key initiatives included in the 2006 National Gender Policy to curb continued spread of gender-based violence [47]. The National Assembly has also enacted Violence Against Persons (Prohibition) Act 2015. This Act provides sufficient legal protection against all forms of gender-based violence and prescribes imprisonments for wide range spousal violence such as forceful ejection from home, forceful isolation from friends/relatives, spouse battery and harmful cultural and widowhood practices [48]. It is expected that the enforcement of the Act will reduce the prevalence of spousal violence in the country.

### Data and sample

Data analysed in the study were based on Women’s individual recode datasets of the 2008 and 2013 Nigeria Demographic and Health Survey (NDHS). The datasets were pooled to make up for the small proportion of childless women in each round of the NDHS. By combining the datasets, the analyses reflect a better fit of the situation than one specific round of the survey. Comprehensive descriptions of the 2008 and 2013 NDHS sample designs have been published elsewhere [49, 50]. The current study did not analyse all women included in the surveys. To be included in the study a woman must have been included in the domestic violence module; be currently married; be aged 35–49 years; not currently pregnant; has never used contraceptives; has non-zero ideal family size, not sterilised at zero parity, and must not have been remarried. Women who did not satisfy the criteria were excluded to reduce bias in the analysis. This criterion was adopted to address some of the methodological issues confronting the measurement of infertility using the DHS data [51]. The study focused on women aged 35–49 years because at this age groups most women in Nigeria have been married for reasonable length of time with the median age of first marriage among women aged 25–49 been 18.1 years as at 2013 [50]. The weighted sample size was 8664 women.

### Outcome variable

The outcome variable was spousal violence. In the NDHS, three key types of spousal violence were measured, namely physical, sexual, and emotional violence. To derive physical violence in the surveys, women were asked to affirm whether their last husband/current partner ever: push, shake, or throw something; slap; twist arm or pull hair; punch with fist or with something else; kick, drag, or beat up; tried to choke or burn; threaten or attack with a knife, gun, or any other weapon at them to purposively hurt them. To derive sexual violence in the surveys, women were asked whether their last husband/current partner ever physically forced them to have sex or perform other sexual acts when they do not desire such acts. To derive emotional violence in the surveys, women were asked whether their last husband/current partner ever said or did something to humiliate them in the presence of others; threaten to hurt or harm them or someone close to them; or insulted or make them feel bad about themselves [50]. In the current study, indicators of specific type of spousal violence were combined to form two categories, namely women who had never experienced the specific violence, and women who had ever experienced at least one type of the specific violence. The three types of spousal violence were then combined into a single spousal violence variable with binary outcomes of ever or never experienced at least one type of spousal violence. The outcome of interest in the study was ever experienced spousal violence.

### Explanatory and control variables

The main explanatory variable was parity which was categorised into three, namely, childless (never had a live birth), ever born only one child, and ever born two or more children. In addition to the main explanatory variable, three sets of variables were included in the analysis. Four individual characteristics, namely current age, education, age at first marriage, and pregnancy termination were selected to associate spousal violence with individual characteristics. These variables have been associated with spousal violence in previous studies [21, 25, 39, 52, 53]. Four community level variables namely, community attitude to wife-battery, community level of family-of-origin violence (intergenerational exposure to family violence), community residence type, and geographic region were included to associate spousal violence with community characteristics. Previous studies had established the relevance of these variables to spousal violence [38, 52]. With exclusions of community residence type and geographic region, the other community variables were generated from individual characteristics. Community attitude to wife-battery was generated from individual response to attitude to wife-battery if wife refuse to have sex with husband, while community level of family-of-origin violence was generated from individual response to witnessing interparental violence (father ever beat mother).

The community variables were generated through method of aggregation. This involve determining a benchmark to indicate proportion of women who had a specific attribute in the community, and then aggregate the variable at the cluster (community) level. The proportions of women having the attribute were then ranked and divided into two groups to show low and high proportions of the attribute. This method has been widely used in studies exploring community variables [38, 52]. Four household variables were selected for statistical control in the study. These are partner alcoholic consumption, power relation in the family, type of marriage, and household wealth. The selection of these variables was guided by literature on risk factors for spousal violence [54–56]. Power relation in the family was based on women’s participation in three household decision, namely, own health care, large household purchase, and visits to friends/relatives. Power relation in families in which the male partner solely takes the decisions were defined as male dominated, while others were defined as not male dominated. Women were grouped as being in ‘polygynous’ or ‘monogamous’ unions if their male partners had at least one other wife or not. Though, the practice of polygyny has reduced from 41% in 1990 to 33% in 2013 [57, 50], it remains an important marital practice that could impact spousal violence in the country. The Variance Inflation Factor (VIF) with mean VIF of 2.41 confirmed the absence of substantial multicollinearity among the explanatory variables.

### Statistical analyses

Statistical analyses were performed at three levels. At first level, descriptive statistics, namely frequency distribution and percentages were used to describe respondents’ demographic, health and spousal violence profile. At the second level, simple cross tabulation of explanatory variables and spousal violence was performed to determine prevalence of spousal violence by each explanatory variable. The unadjusted binary logit coefficient was used to examine the relationship between the explanatory and outcome variables. Positive regression coefficient indicates positive relationship and negative regression coefficient indicate negative relationship. At the third level, the multilevel mixed-effects logistic regression analysis was applied for three reasons. Firstly, complex surveys such as the NDHS were hierarchical in nature and suitable for hierarchical analytical methods. Secondly, studies have shown that the causes of spousal violence go beyond individual level [38, 52, 56]. Thirdly, multilevel analysis is consistent with the ecological framework adopted in the study.

The multilevel model specified was a 2-level model (individual and community). The mathematical equation of the model was expressed as:

$$ {y}_{ij}={\beta}_0{X}_{oij}+{\beta}_1{X}_{1 ij}+{U}_j{X}_{oij}+{\varepsilon}_{ij} $$

where:

*y*_*ij*_ is spousal violence of the i ^th^ woman in the j ^th^ community (cluster).

*β*_0_, *β*_1_ are the fixed component of the model.

*U*_*j*_, *ε*_*ij*_ are the random component of the model. The Stata 12 *xtmelogit* command [58] was used to estimate the model parameters in four nested models. Model 1 included only parity while Model 2 was based on parity and the four selected individual variables. Model 3 was based on parity and the four selected community variables. Model 4 was the full model which included all explanatory and control variables.

The fixed-effects of the model were measured by odds ratios of binary logistic regression. The random-effects of the model were measured by the Intra-Class Correlation (ICC) calculated as: $$ \frac{\sigma_u^2}{\sigma_u^2+\frac{\pi^2}{3}} $$ [59], where $$ {\sigma}_u^2 $$ is the variance at the community level and $$ \raisebox{1ex}{${\pi}^2$}\!\left/ \!\raisebox{-1ex}{$3$}\right. $$is equal to 3.29. The ICC expressed in percentage show the variation in spousal violence due to community characteristics. The models were diagnosed using the Log-likelihood Ratio test (LR test) and the Akaike’s Information Criterion (AIC). The LR test usually compares each fitted model with one-level ordinary linear regression. The result of the test will indicate if the fitted model is adequate for the data being analysed or not. AIC is expected to reduce in size as more variables are been added to each successive model. This also confirms the goodness-of-fit of the fitted model. The statistical significance for all tests was set at *p* < 0.05. All statistical analyses were performed using Stata 12.

### Ethics statement

Data analysed in the study were formally requested from MEASURE DHS. Authorisation was granted. The respondents were anonymous. No steps have been taken to identify individuals and communities included in the surveys. Hence, the analyses are not in any way injurious to any individual, household or community.

## Results

### Univariate results

Table 1 present respondents’ demographic, health and spousal violence profile. Majority of the respondents have two or more children. Only 2.8% of respondents are childless, while 2.9% of respondents have only one child. Women aged 35–39 years were dominant among respondents. Slightly more than half of respondents do not have formal education. Primary education was the dominant educational level attained among respondents who had educational attainments. The dominant age group at first marriage was age 15–19 years, but slightly more than a quarter were married at ages less than 15 years. Majority of respondents have never experienced a pregnancy termination, but more than one-tenth had experienced at least one pregnancy termination. More than half of respondents live in communities where the proportion of women who justified wife-battery was low, while nearly half of them live in communities where the proportion of women who justified wife-battery was high.

**Table 1:** Respondents’ demographic, health and spousal violence profile, Nigeria, 2008–2013

Characteristic	Frequency (*n* = 8664)	Percentage	Characteristic	Frequency (*n* = 8664)	Percentage
Parity			Household characteristic:		
Childless	239	2.8	Type of marriage		
Only one child	250	2.9	Monogamy	5455	63.0
Two or more children	8175	94.3	Polygyny	3209	37.0
Maternal age (years)			Partner alcoholic consumption		
35–39	3416	39.4	Does not drink	6946	80.2
40–44	2725	31.5	Drinks	1718	19.8
45–49	2523	29.1	Household wealth		
Maternal education			Poorest	1934	22.3
None	4349	50.2	Poorer	1683	19.4
Primary	1932	22.3	Middle	1631	18.8
Secondary	1654	19.1	Richer	1607	18.6
Higher	729	8.4	Richest	1809	20.9
Pregnancy termination			Family power relation		
Ever experienced	1428	16.5	Not male dominated	3318	38.3
Never experienced	7236	83.5	Male dominated	5346	61.7
Age at first marriage			Community characteristic: Attitude to wife-battery (Proportion justified wife-battery)		
Less than 15 years	2316	26.7			
15–19 years	3335	38.5			
20–24 years	1794	20.7	Low	4453	51.4
25 years or older	1219	14.1	High	4211	48.6
Spousal physical violence			Family-of-origin violence: (Proportion witnessed interparental violence)		
Never experienced	7825	90.3			
Ever experienced	839	9.7	Low	4522	52.2
Spousal sexual violence			High	4142	47.8
Never experienced	8435	97.4	Community residence type		
Ever experienced	229	2.6	Urban	3328	38.4
Spousal emotional violence			Rural	5336	61.6
Never experienced	7142	82.4	Geographic region		
Ever experienced	1522	17.6	North-central	1157	13.4
Spousal violence (at least one type)			North-east	1220	14.1
Never experienced	6804	78.5	North-west	2906	33.5
Ever experienced	1860	21.5	South-east	1023	11.8
			South-south	916	10.6
			South-west	1442	16.6
Total	8664	100.0	Total	8664	100.0

Source: Authors’ analysis based on 2008 and 2013 pooled data from Nigeria Demographic and Health Survey

Likewise, more than half of respondents live in communities where women who witnessed interparental violence was low compared with nearly half of respondents who live in communities where the proportion was high. Women who reside in rural communities were dominant among respondents. Respondents from the north-west region of the country were dominant in the sample. Monogamy was the dominant type of marital union among respondents, but more than one-third of respondents were in polygynous unions. Majority of respondents’ partners does not drink alcohol. The power relation in nearly two-thirds of respondents’ family was male dominated. Household wealth quintile among respondents was nearly evenly distributed, but higher proportion of respondents live in ‘poorest’ households. The least spousal violence experienced by respondents was spousal sexual violence. Nearly one-tenth of respondents had experienced at least one type of spousal physical violence, while nearly one-fifth of respondents had experienced at least one type of spousal emotional violence. Overall, 21.5% of respondents had experienced at least one type of spousal violence.

### Bivariate results

Table 2 present the bivariate relationship between the explanatory and outcome variables. Parity and spousal violence were positively associated with higher prevalence of spousal violence among women who have children. Current age and spousal violence were negatively associated. The prevalence of spousal violence was nearly evenly distributed across the age groups except age group 45–49 years. The relationship between maternal education and spousal violence was mixed. At lower educational levels, the relationship was positive, but at higher educational level, the relationship was negative. Likewise, age at first marriage had a mixed relationship with spousal violence. At lower ages, the relationship was positive while it was negative at age 25 years or older. Pregnancy termination and spousal violence were negatively associated with higher prevalence of spousal violence among women who had experienced a pregnancy termination. Community attitude to wife-battery and spousal violence were positively associated with higher prevalence of spousal violence in communities with high proportion of women justifying wife-battery. Community level of family-of-origin violence was positively associated with spousal violence with higher prevalence of spousal violence in communities where high proportion of women witnessed interparental violence.

**Table 2:** Unadjusted binary logistic regression showing bivariate relationship between explanatory variables and spousal violence

Characteristic	% of spousal violence	Coeff.	P > |t|	95% CI	Characteristic	% of spousal violence	Coeff.	*P* > |t|	95% CI
Parity									
Childless^ref^	12.2	–	–	–	Community residence type				
Only one child	16.6	0.360	0.212	−0.204 0.923	Urban^ref^	20.7	–	–	–
Two or more children	21.9	0.704	0.002	0.260 1.148	Rural	22.0	0.074	0.473	−0.129 0.278
Maternal age (years)					Geographic region				
35–39 ^ref^	22.2	–	–	–	North-central^ref^	26.6	–	–	–
40–44	21.7	−0.029	0.743	−0.202 0.144	North-east	28.3	0.085	0.557	−0.198 0.368
45–49	20.2	−0.121	0.147	−0.284 0.043	North-west	15.4	−0.686	<0.001	−0.989 -0.383
Education					South-east	30.6	0.195	0.209	−0.109 0.500
None ^ref^	18.1	–	–	–	South-south	23.2	−0.184	0.251	−0.497 0.130
Primary	28.1	0.568	<0.001	0.381 0.754	South-west	16.1	−0.636	<0.001	−0.919 -0.353
Secondary	25.1	0.416	<0.001	0.216 0.615	Type of marriage				
Higher	15.6	−0.183	0.234	−0.484 0.118	Monogamy^ref^	21.4	–	–	–
Age at first marriage					Polygyny	21.5	0.007	0.930	−0.147 0.161
Less than 15 years ^ref^	20.3	–	–	–	Partner alcoholic consumption				
15–19	21.1	0.048	0.597	−0.129 0.224	Does not drink^ref^	17.3	–	–	–
20–24	25.1	0.273	0.006	0.077 0.469	Drinks	38.5	1.100	<0.001	0.935 1.266
25 years or older	19.5	−0.046	0.677	−0.263 0.171	Family power relation				
Pregnancy termination					Not male dominated^ref^	23.8	–	–	–
Ever terminated ^ref^	27.1	–	–	–	Male dominated	20.0	−0.221	0.006	−0.379 -0.062
Never terminated	20.3	−0.376	<0.001	−0.548 -0.204	Household wealth				
Proportion justifying wife-battery					Poorest^ref^	18.0	–	–	–
Low ^ref^	18.5	–	–	–	Poorer	24.6	0.400	<0.001	0.176 0.624
High	24.6	0.358	<0.001	0.261 0.634	Middle	24.4	0.386	0.002	0.138 0.635
Proportion who witnessed interparental violence					Richer	23.1	0.314	0.014	0.065 0.564
Low ^ref^	17.9	–	–	–	Richest	18.1	0.011	0.941	−0.273 0.294
High	25.4	0.447	<0.001	0.261 0.634					

Notes: ref. (reference category)

Community residence type and spousal violence were positively associated with slightly higher prevalence of spousal violence in rural communities. The relationship between geographic region and spousal violence was mixed with positive relationship in the north-east and south-east regions, while the relationship was negative in other zones. Type of marital union and spousal violence were positively associated with nearly the same level of prevalence irrespective of type of marriage. Partner alcoholic consumption was positively associated with spousal violence with higher prevalence of spousal violence among women whose male partners’ drinks alcohol. Power relation within the family and spousal violence were negatively associated. However, higher prevalence of spousal violence was reported among women in households that are not male dominated. The prevalence of spousal violence increases as household wealth improves from ‘poorest’ to ‘poorer’, but thereafter reduce consistently as household wealth improves showing positive relationship between household wealth and spousal violence.

### Multivariate results

Table 3 present the fixed-effects of the multilevel regression. In Model 1 which included only the main explanatory variable, women who have two or more children were 57% more likely to experienced spousal violence compared with childless women (OR = 1.570: CI: 1.074–2.294). When the individual characteristics were included in Model 2, women who have two or more children were 52.3% more likely to experienced spousal violence compared with childless women (OR = 1.523; CI: 1.037–2.247). In the model, women aged 45–49 years were 13.9% less likely to experience spousal violence compared with women aged 35–39 years (OR = 0.861; CI: 0.751–0.988). The likelihood of experiencing spousal violence reduces as maternal education improves. Women with higher education were 24.6% less likely to experience spousal violence compared with uneducated women (OR = 0.754; CI: 0.591–0.961). Women who had never terminated a pregnancy were 29.3% less likely to experienced spousal violence compared with women who had terminated a pregnancy (OR = 0.707; CI: 0.613–0.816). In Model 3, women who have two or more children were 67.2% more likely to experience spousal violence compared with childless women (OR = 1.672; CI: 1.140–2.452). In the model, the likelihood of spousal violence was 46.2% higher among women in communities with high proportion of women who justified wife-battery compared with women in communities with low proportion (OR = 1.462; CI: 1.245–1.718); and the likelihood of spousal violence was 51.0% higher among women in communities with high proportion of women who witnessed interparental violence compared with women in communities with low proportion (OR = 1.510; CI: 1.294–1.761).

**Table 3:** Fixed-effects of multilevel logistic regression showing influence of explanatory variables on likelihood of spousal violence

Characteristic	Model 1		Model 2		Model 3		Model 4	
	Odds Ratio	95% CI	Odds Ratio	95% CI	Odds Ratio	95% CI	Odds Ratio	95% CI
Parity								
Childless^ref^	1.000	–	1.000	–	1.000	–	1.000	–
Only one child	1.293***	0.790 2.114	1.250***	0.762 2.051	1.295***	0.789 2.126	1.223***	0.739 2.023
Two or more children	1.570**	1.074 2.294	1.523**	1.037 2.247	1.672**	1.140 2.452	1.508**	1.017 2.234
Maternal age								
35–39 years^ref^			1.000	–			1.000	–
40–44 years			0.927***	0.813 1.056				
45–49 years			0.861**	0.751 0.988				
Education								
None^ref^			1.000	–			1.000	–
Primary			1.566*	1.351 1.816				
Secondary			1.285**	1.086 1.519				
Higher			0.754**	0.591 0.961				
Age at first marriage								
Less than 15 years^ref^			1.000	–			1.000	–
15–19 years			1.037***	0.893 1.203				
20–24 years			1.238**	1.041 1.471				
25 years or older			1.087***	0.887 1.332				
Pregnancy termination								
Ever terminated^ref^			1.000	–			1.000	–
Never terminated			0.707*	0.613 0.816				
Proportion who justified wife-battery								
Low					1.000	–	1.000	–
High					1.462*	1.245 1.718	1.425*	1.216 1.671
Proportion who witnessed interparental violence								
Low					1.000	–	1.000	–
High					1.510*	1.294 1.761	1.359*	1.167 1.582
Community residence type								
Urban^ref^					1.000	–	1.000	–
Rural					1.125***	0.979 1.292	1.005***	0.855 1.182
Geographic region								
North-central^ref^					1.000	–	1.000	–
North-east					1.077***	0.879 1.319	1.274**	1.120 1.590
North-west					0.314*	0.326 0.478	0.544*	0.439 0.676
South-east					1.157***	0.911 1.471	0.792***	0.618 1.014
South-south					0.770**	0.600 0.988	0.540*	0.417 0.698
South-west					0.528**	0.411 0.676	0.479*	0.372 0.616
Type of marriage								
Monogamy^ref^							1.000	–
Polygyny							1.276*	1.125 1.448
Partner alcoholic consumption								
Does not drink^ref^							1.000	–
Drinks							2.952*	2.563 3.400
Family power relation								
Not male dominated^ref^							1.000	–
Male dominated							0.879**	0.774 0.998
Household wealth								
Poorest^ref^							1.000	–
Poorer							1.101***	0.915 1.323
Middle							1.058***	0.861 1.301
Richer							0.935***	0.737 1.188
Richest							0.771***	0.580 1.024

Ref. (reference category); *p < 0.001, **p < 0.05, ***p > 0.05 (not significant)

In the full model (Model 4), women who have two or more children were 50.8% more likely to experienced spousal violence compared with childless women (OR = 1.508; CI: 1.017–2.234). In the model, three variables, namely, age at first marriage, community residence type, and household wealth reveal no significant association with spousal violence. Other variables reveal varying degrees of association with spousal violence. Women aged 45–49 years were 14.8% less likely to experience spousal violence compared with women aged 35–39 years (OR = 0.852; CI: 0.740–0.982). The likelihood of spousal violence increases with maternal education but reduces at higher education though this was without statistical significance. The likelihood of spousal violence was 26.4% less likely among women who had never terminated pregnancy compared with women who had ever terminated pregnancy (OR = 0.736; CI: 0.634–0.853).

The likelihood of spousal violence was 42.5% higher among women who live in communities with high proportion of women who justified wife-battery compared with women in communities with low proportion of women who justified wife-battery (OR = 1.425; CI: 1.216–1.671). Likewise, the likelihood of spousal violence was 35.9% more likely in communities with high proportion of women who witnessed interparental violence compared with women in communities with low proportion of witnessing interparental violence (OR = 1.359; CI: 1.167–1.582). The likelihood of spousal violence was higher only in the north-east region compared with other regions in the country. The likelihood of spousal violence was 27.6% higher among women in polygynous unions compared with women in monogamous unions (OR = 1.276; CI: 1.125–1.448). Women whose male partner drinks alcohol were nearly three times more likely to experience spousal violence compared with women whose male partners does not drink alcohol (OR = 2.952; CI: 2.563–3.400).

Table 4 present the random effects on spousal violence. The consistent decline in the values of the log-likelihood and the AIC suggest that the models were adequately fitted. The LR test at each model confirms the goodness-of-fit of the models. Results in all the models provide evidence that community characteristics are part of the causes of spousal violence. In Model 1, the community variables accounted for 15.3% of the variation in spousal violence. Though, this proportion declines to 14.4% in Model 2 and to 11.1% in Model 4, but it provided evidence that community characteristics are indeed associated with spousal violence.

**Table 4:** Random-effects of multilevel logistic regression showing community-level influence on the likelihood of spousal violence

Parameter	Empty Model	Model 1	Model 2	Model 3	Model 4
Community-level variance	0.596	0.593	0.555	0.477	0.412
Log-likelihood	−4487.5	−4483.9	−4434.4	−4358.6	−4193.1
LR test	LR χ2 = 206.8; *p* < 0.001	LR χ2 = 205.2; *p* < 0.001	LR χ2 = 180.5; *p* < 0.001	LR χ2 = 133.6; *p* < 0.001	LR χ2 = 96.3; *p* < 0.001
ICC (%)	15.3%	15.3%	14.4%	12.7%	11.1%
AIC	8980.9	8977.8	8896.8	8743.2	8444.1

## Discussion

This study compared the likelihood of spousal violence among childless women and women who have children using pooled data of 2008 and 2013 NDHS. This is probably the first Nigerian study that used the NDHS to investigate the likelihood of spousal violence among these categories of women. The high quality of the NDHS as well as its national representativeness makes the result a true reflection of the childlessness-spousal violence scenario in Nigeria. The study improves upon the limitations of previous Nigerian studies conducted among only infertile women attending fertility clinics [23, 24]. It made contributions to existing knowledge by providing research evidence on the Nigerian side of childlessness-spousal violence situation on which mixed findings currently exists [22, 37–39]. The 2.8% proportion of childless women found in the study is comparable to the 2.3% reported in an earlier study [39] but lower than the 8.5% reported and the 10.8% reported by two previous studies [22, 38]. The possible reason for these variations is the different age groups studied. In the current study, we excluded younger women which reduce the proportion of childless women in the sample. The 21.5% prevalence of at least one type of spousal violence found in the study is within the range of spousal violence reported across the world [26, 31]. Three key issues emerged from the study findings.

Firstly, the likelihood of spousal violence is lower among childless women in Nigeria. In a country where fertility remains a cultural requirement, it is likely that this finding may spur further investigation among researchers in the country. The lower likelihood of spousal violence found among childless women contradicts findings in two Indian studies [22, 39] but agrees with finding in an earlier study [38], and to a large extent consistent with finding in another earlier study [37] which reported that both childless women and women with high parity had elevated risk of spousal violence. It is important to emphasise that while it is true that childlessness make individuals and couples unhappy, childlessness may not be a sufficient reason for violence by an intimate partner. There is evidence of spousal violence against pregnant women across the world [31, 32] which not only negates violence due to childlessness, but also suggests that there are other issues beyond being childless that could lead to fmay have to a large extent reduces co-wives in an earlier study [30] that pregnancy was a stimulus of domestic violence is particularly important for understanding that childlessness may not necessarily lead to spousal violence.

There are at least four reasons to support lower likelihood of spousal violence found among childless women in the study. One, the declining prevalence of polygyny in the country [57, 50] may have to a large extent reduces co-wives competition for children in the family which is one of the factors that could promote violence between the male partner and a childless female partner. Two, the less attention now paid to agriculture in many communities in the country may have also weakened cultural and communal demand for children which was mostly based on desire for large number of children to work in the family farm. Third, changing nature of family system where majority of families are now nuclear compared with the extended family system that was previously dominant in many parts of the country may have also contributed to decline in spousal violence due to childlessness. This gives credence to the finding in a previous study [56] that extended family interference was a significant cause of spousal violence. In most cases the perpetrators of violence against childless women are family members and other relatives and not necessarily the intimate partner. A recent study [7] also reported that childlessness was becoming more tolerated in Nigeria, though not yet fully embraced in the country. This also indicates a possible change in cultural perception of the childless woman. Four, children may also be a source of violence between intimate partners as there are possibility that spousal violence could occur when one partner is not responsive to child care or when attention are unduly shifted to children at the expense of one of the partners. It is important for future research in Nigeria to explore the impact of children issues on the prevalence of spousal violence.

Secondly, the causes of spousal violence are rooted in individual, household and community factors. Virtually all variables investigated reveal association with spousal violence. In line with previous findings, the study found that pregnancy termination [53]; justifying wife-battery [52]; exposure to interparental violence [38]; alcoholic consumption [54]; and power relation in the family [55] were significantly associated spousal violence among the women. These findings not only demonstrate the relevance of the ecological model to addressing the issue of spousal violence, it also suggest that current multilevel interventions such as the BCC programme targeting individuals, households and communities in the country may go a long way in reducing extent of intimate partner violence in the country if effectively implemented. However, the implementation should be strengthened by adequate enforcement of the newly enacted Violence Against Persons (Prohibition) Act of 2015. There is need to boost awareness of the Act and its provisions through comprehensive public dissemination. The Act could be made available to all public institutions, schools and Marriage Registries in the country.

Thirdly, widespread unavailability and lack of access to assisted reproductive technologies used to address fertility challenges should be regarded as a violation of the fundamental rights of individuals and couples who desire to have children but are further incapacitated by lack of access to these facilities. All existing population and health policies in the country are based on the principle that all citizens of the country have a right to healthy and productive life [43, 44] and that governments at all tiers of the federation will provide essential health services to enable individuals meet their reproductive health needs. It is therefore important that health delivery facilities be expanded in the country. Though such expansion will require more public health funding, governments in the country could devise additional ways of raising fund through more partnerships with the private sector and health development partners.

Analyses carried out in the study suffer from some drawbacks. Both the outcome and explanatory variables are measured at the moment of the surveys. It is not plausible to assume that the same level of parity or spousal violence reported in the surveys remain constant over time. Findings in the study are therefore limited by the cross-sectional nature of the data analysed. The models estimated in the study could not have capture all known risk factors of spousal violence at both individual and community levels. Future studies on the subject matter may focus on variables excluded in the current study. In addition, findings in the study have not been augmented with qualitative data because the goal of the research was mainly to determine whether the likelihood of spousal violence was lower or higher among childless women compared with women who have children, which does not require the collection of qualitative data. However, in-depth understanding of how childlessness interacts with other social factors to affect spousal violence may be revealed in a qualitative study. A follow-up study is being planned to explore this aspect.

## Conclusions

The study provided research evidence that the likelihood of spousal violence was lower among childless women in Nigeria. The findings support some earlier results while contradicting others. The study submits that there are other issues cutting across individual, household and community factors that cause spousal violence. Adequate enforcement of the newly enacted Violence Against Persons (Prohibition) Act of 2015 will go a long way to boost existing BCC programme aiming to reduce the prevalence of spousal violence in the country. There is need to expand health delivery facilities in the country to make assisted reproductive technologies more accessible to individual and couples facing fertility challenges.

## Abbreviations

DHS: Demographic and Health Survey
FMoH: Federal Ministry of Health
NDHS: Nigeria Demographic and Health Survey
NPC: National Population Commission

## References

[1] Caldwell JC, Caldwell P (1987). The cultural context of high fertility in sub-Saharan Africa. Popul Dev Rev.

[2] Jeon Y, Rhyu S-Y, Shields MP (2010). Fertility in sub-Saharan African countries with consideration to health and poverty. Afr Dev Rev.

[3] Bankole A, Audam S (2011). Fertility preferences and contraceptive use among couples in sub-Saharan Africa. Afr Popul Stud.

[4] Bongaarts J, Cleland J, Townsend JW, Bertrand JT, Gupta MD (2012). Family planning programs for the 21ST century.

[5] Tabong PT-N, Adongo PB. Infertility and childlessness: a qualitative study of the experiences of infertile couples in northern Ghana. BMC Pregnancy Childbirth. 2013;13:72. https://bmcpregnancychildbirth.biomedcentral.com/articles/10.1186/1471-2393-13-72. 10.1186/1471-2393-13-72 PMC3610195 23517021

[6] Dimka RA, Dein SL (2013). The work of a woman is to give birth to children: cultural constructions of infertility in Nigeria. Afr J Reprod Health.

[7] Ibisomi L, Mudege NN (2014). Childlessness in Nigeria: perceptions and acceptability. Cult Health Sex.

[8] Larsen U, Hollos M, Obono O, Whitehouse B (2010). Suffering infertility: the impact of infertility on Women’s life experiences in two Nigerian communities. J Biosoc Sci.

[9] Makinde OA, Olaleye O, Makinde OO, Huntley SS, Brown B. Baby factories in Nigeria: starting the discussion toward a National Prevention Policy. Trauma Violence Abuse. 2015:1–8. 10.1177/1524838015591588. 10.1177/1524838015591588 26209095

[10] Hammarberg K, Kirkman M (2013). Infertility in resource-constrained settings: moving towards amelioration. Reprod BioMed Online.

[11] Rutstein SO, Shah IH (2004). Infecundity, infertility, and childlessness in developing countries. DHS comparative reports no. 9.

[12] Bolvin J, Bunting L, Collins JA, Nygren KG (2007). International estimates of infertility prevalence and treatment-seeking: potential need and demand for infertility medical care. Hum Reprod.

[13] Mascarenhas MN, Flaxman SR, Boerma T, Vanderpoel S, Stevens GA (2012). National, regional, and global trends in infertility prevalence since 1990: a systematic analysis of 277 health surveys. PLoS Med.

[14] Araoye MO (2003). Epidemiology of infertility: social problems of the infertile couples. West Afr J Med.

[15] Ahmed A, Bello A, Mbibu NH, Maitama HY, Kalayi GD (2010). Epidemiological and aetiological factors of male infertility in Northern Nigeria. Niger J Clin Pract.

[16] Ombelet W, Cooke I, Dyer S, Serour G, Devroey P (2008). Infertility and the provision of infertility medical services in developing countries. Hum Reprod Update.

[17] Luk BH-K, Loke AY (2015). The impact of infertility on the psychological well-being, marital relationships, sexual relationships, and quality of life of couples: a systematic review. Sex Marital Ther.

[18] Upkong D, Orji EO (2006). Mental health of infertile women in Nigeria. Turk Psikiyatri Derg.

[19] Umezulike AC, Efetie ER (2004). The psychological trauma of infertility in Nigeria. Int J Gynaecol Obstet.

[20] Iris A, Kirmizi DA, Taner CE (2013). Effects of infertility and infertility duration on female sexual functions. Arch Gynecol Obstet.

[21] Yildizhan R, Adali E, Kolusari A, Kurdoglu M, Yildizhan B, Sahin G (2009). Domestic violence against infertile women in a Turkish setting. Int J Gynaecol Obstet.

[22] Pasi AL, Hanchate MS, Pasha MA (2011). Infertility and domestic violence: cause, consequence and management in Indian scenario. BIOMED RES-INDIA.

[23] Aduloju PO, Olagbuji NB, Olofinbiyi AB, Awoleke JO (2015). Prevalence and predictors of intimate partner violence among women attending infertility clinic in south-western Nigeria. Eur J Obstet Gynaecol Reprod Biol.

[24] Iliyasu Z, Galadanci HS, Abubakar S, Auwal MS, Odoh C, Salihu HM (2016). Et. Phenotypes of intimate partner violence among women experiencing infertility in Kano, Northwest Nigeria. Int J Gynaecol Obstet.

[25] Ozgoli G, Sheikhan Z, Zahiroddin A, Nasiri M, Amiri S, Badr FK (2016). Evaluation of the prevalence and contributing factors of psychological intimate partner violence in infertile women. J midwifery Reprod Health.

[26] Kishor S, Johnson K (2004). Profiling domestic violence: a multi-country study.

[27] Devries K, Watts C, Yoshihama M, Kiss L, Schraiber LB, Deyessa N (2011). Et al. violence against women is strongly associated with suicide attempts: evidence from the WHO multi-country study on women’s health and domestic violence against women. Soc Sci Med.

[28] Jina R, Thomas LS (2013). Health consequences of sexual violence against women. Best Pract Res Clin Obstet Gynaecol.

[29] Pereira AR, Vieira DN, Magalhães T (2013). Fatal intimate partner violence against women in Portugal: a forensic medical national study. J Forensic Legal Med.

[30] Burch RL, Gallup GG (2004). Pregnancy as a stimulus for domestic violence. J Fam Violence.

[31] Devries KM, Kishor S, Johnson H, Stöckl H, Bacchus LJ, Garcia-Moreno C (2010). Intimate partner violence during pregnancy: analysis of prevalence data from 19 countries. Reprod Health Matters.

[32] Bessa MMM, Drezett J, Rolim M, Carlos de Abreu L (2014). Violence against women during pregnancy: sistematized revision. Reprod Clim.

[33] Ardabily HE, Moghadam ZB, Salsali M, Ramezanzadeh F, Nedjat S (2011). Prevalence and risk factors for domestic violence against infertile women in an Iranian setting. Int J Gynaecol Obstet.

[34] Uwaoma NC, Osita-Njoku A, Madukwe AU (2012). Education, male child and childlessness as predictors of spouse abuse among rural and urban Igbo-Nigerian women. CSCanada.

[35] Stellar C, Garcia-Moreno C, Temmerman M, van der Poel S. A systematic review and narrative report of the relationship between infertility, subfertility, and intimate partner violence. Int J Gynaecol Obstet. 2016;133:3–8. http://onlinelibrary.wiley.com/doi/10.1016/j.ijgo.2015.08.012/pdf. 10.1016/j.ijgo.2015.08.012 26797197

[36] Hollos M (2003). Profiles of infertility in southern Nigeria: Women's voices from Amakiri. Afr J Reprod Health.

[37] McCloskey LA, Williams C, Larsen U (2005). Gender inequality and intimate partner violence among women in Moshi, Tanzania. Int Fam Plan Perspect.

[38] Koenig MA, Stephenson R, Ahmed S, Jejeebhoy SJ, Campbell J (2006). Individual and contextual determinants of domestic violence in North India. Am J Public Health.

[39] Syamala TS (2012). Infertility in India: levels, trends, determinants and consequences. Working paper 284.

[40] McLeroy KR, Bibeau D, Steckler A, Glanz K (1988). An ecological perspective on health promotion programs. Health Educ Q.

[41] Population Reference Bureau. 2015 World Population Data Sheet with a special focus on women empowerment. http://www.prb.org/pdf15/2015-world-population-data-sheet_eng.pdf. Accessed 18 Mar 2016.

[42] Adegbola O, Akindele MO (2013). The pattern and challenges of infertility management in Lagos, Nigeria. Afr Health Sci.

[43] National Population Commission (2004). National Policy on Population for Sustainable Development.

[44] Federal Ministry of Health. Revised National Health Policy. Abuja, Nigeria: FMoH; 2004. http://cheld.org/wp-content/uploads/2012/04/Nigeria-Revised-National-Health-Policy-2004.pdf. Accessed May 18 2016.

[45] Federal Ministry of Health. National Family Planning/Reproductive Health Service Protocols. (Revised edition). Abuja, Nigeria: FMoH;2010.

[46] Omosun AO, Kofoworola O. Knowledge, attitude and practice towards child adoption amongst women attending infertility clinics in Lagos State, Nigeria. Afr J Prm Health Care Fam Med. 2011;3(1), Art. #259, 8 pages. doi:10.4102/phcfm.v3i1.259.

[47] Federal Ministry of Women Affairs and Social Development. National Gender Policy. Abuja, Nigeria: FMWA & SD;2006.

[48] Onyemelukwe C. Legislating on Violence Against Women: A Critical Analysis of Nigeria’s Recent Violence Against Persons (Prohibition) Act. 5 DePaul J. Women. Gender & L. 2015;2016. http://via.library.depaul.edu/cgi/viewcontent.cgi?article=1028&context=jwgl. Accessed Oct 6 2016.

[49] National Population Commission (NPC) [Nigeria] & ICF Macro. (2009). Nigeria demographic and health survey 2008. Abuja, Nigeria: National Population Commission and ICF Macro. https://www.dhsprogram.com/pubs/pdf/FR222/FR222.pdf. Accessed July 21 2016.

[50] National Population Commission (NPC) [Nigeria] & ICF International. (2014). Nigeria Demographic and Health Survey 2013. Abuja, Nigeria, and Rockville, Maryland, USA: NPC and ICF International. https://www.dhsprogram.com/pubs/pdf/FR293/FR293.pdf. Accessed July 19 2016.

[51] Mascarenhas MN, Cheung H, Mathers CD, Stevens GA. Measuring infertility in populations: constructing a standard definition for use with demographic and reproductive health surveys. Popul Health Metr. 2012;10:17. https://pophealthmetrics.biomedcentral.com/articles/10.1186/1478-7954-10-17. 10.1186/1478-7954-10-17 PMC3511253 22938182

[52] Antai D, Adaji S. Community-level influences on women's experience of intimate partner violence and terminated pregnancy in Nigeria: a multilevel analysis. BMC Pregnancy Childbirth. 2012;12:128. https://bmcpregnancychildbirth.biomedcentral.com/articles/10.1186/1471-2393-12-128. 10.1186/1471-2393-12-128 PMC3541204 23150987

[53] Hall M, Chappell LC, Parnell BL, Seed PT, Bewley S (2014). Associations between intimate partner violence and termination of pregnancy: a systematic review and meta-analysis. PLoS Med.

[54] Foran HM, O'Leary KD (2008). Alcohol and intimate partner violence: a meta-analytic review. Clin Psychol Rev.

[55] Okenwa LE, Lawoko S, Jansson B (2009). Exposure to intimate partner violence amongst women of reproductive age in Lagos, Nigeria: prevalence and predictors. J Fam Viol.

[56] Clark CJ, Silverman JG, Shahrouri M, Everson-Rose S, Groce N (2010). The role of the extended family in women’s risk of intimate partner violence in Jordan. Soc Sci Med.

[57] Federal Office of Statistics, IRD Macro International. Nigeria Demographic and Health Survey 1990. Lagos and Columbia MD, USA; FOS & IRD/Macro; 1992. https://www.dhsprogram.com/pubs/pdf/FR27/FR27.pdf. Accessed 9 Oct 2016.

[58] StataCorp. Stata: Release 12. Statistical Software. College Station, TX: StataCorp LP; 2011.

[59] Merlo J, Wagner P, Ghith N, Leckie G (2016). An original stepwise multilevel logistic regression analysis of discriminatory accuracy: the case of Neighbourhoods and health. PLoS One.

